# Continue, adjust, or stop antipsychotic medication: developing and user testing an encounter decision aid for people with first-episode and long-term psychosis

**DOI:** 10.1186/s12888-018-1707-x

**Published:** 2018-05-22

**Authors:** Yaara Zisman-Ilani, David Shern, Patricia Deegan, Julie Kreyenbuhl, Lisa Dixon, Robert Drake, William Torrey, Manish Mishra, Ksenia Gorbenko, Glyn Elwyn

**Affiliations:** 1 Department of Rehabilitation Sciences, College of Public Health, Temple University, 1700 North Broad St., Philadelphia, PA 19122 USA; 2 Department of Mental Health, Bloomberg School of Public Health, Johns Hopkins University, Baltimore, MD USA; 3 Pat Deegan, PhD & Associates, LLC, Byfield, MA USA; 4 The Department of Psychiatry, University of Maryland School of Medicine, Baltimore, MD USA; 5 VA Capitol Healthcare Network (VISN 5), Mental Illness Research, Education, and Clinical Center (MIRECC), Baltimore, MD USA; 6 Columbia University Medical Center, New York, NY USA; 7 The Dartmouth Institute for Health Policy and Clinical Practice, Geisel School of Medicine at Dartmouth College, Lebanon, NH USA; 8 Department of Psychiatry, Dartmouth-Hitchcock Medical Center, Lebanon, NH USA; 9 Institute for Healthcare Delivery Science, Department of Population Health Science and Policy, Icahn School of Medicine at Mount Sinai, New York, NY USA; 10 New York State Psychiatric Institute, New York, USA

**Keywords:** Shared decision making, Decision aid, Decision support tool, Option grid, Psychosis, Antipsychotic medication

## Abstract

**Background:**

People with psychosis struggle with decisions about their use of antipsychotics. They often want to reduce the dose or stop, while facing uncertainty regarding the effects these decisions will have on their treatment and recovery. They may also fear raising this issue with clinicians. The purpose of this study was to develop and test a shared decision making (SDM) tool to support patients and clinicians in making decisions about antipsychotics.

**Methods:**

A diverse editorial research team developed an Encounter Decision Aid (EDA) for patients and clinicians to use as part of the psychiatric consultation. The EDA was tested using 24 semistructured interviews with participants representing six stakeholder groups: patients with first-episode psychosis, patients with long-term psychosis, family members, psychiatrists, mental health counselors, and administrators. We used inductive and deductive coding of interview transcripts to identify points to revise within three domains: general impression and purpose of the EDA; suggested changes to the content, wording, and appearance; and usability and potential contribution to the psychiatric consultation.

**Results:**

An EDA was developed in an iterative process that yielded evidence-based answers to five frequently asked questions about antipsychotic medications. Patients with long-term psychosis and mental health counselors suggested more changes and revisions than patients with first-episode psychosis and psychiatrists. Family members suggested more revisions to the answers about potential risks of stopping or adjusting antipsychotics than other respondents.

**Conclusions:**

The EDA was perceived as potentially useful and feasible in psychiatric routine care, especially if presented during the consultation.

**Electronic supplementary material:**

The online version of this article (10.1186/s12888-018-1707-x) contains supplementary material, which is available to authorized users.

## Background

People with serious mental illness, such as schizophrenia, face decisions to continue, reduce or discontinue antipsychotic medications daily. Many fail to formally consult their psychiatrists as part of this decision process. Few prescribers explicitly address this issue with patients during the clinical encounter [1–3]. This situation illuminates two important points to consider in clinical practice. First, it raises concerns about the effectiveness of antipsychotic medications as a primary approach for preventing relapse in psychosis [4]. Second, people with serious mental illness make treatment decisions about their use of medications independently, often without enough information gained in consultation with psychiatrists and other mental health providers about the likely risks and benefits [5–7].

Many studies have addressed the benefits and risks of continuing antipsychotics [8–13] as well as the effects of nonadherence to antipsychotic medications and its antecedents, factors, and outcomes [3, 14, 15]. However, few have focused on the decision-making process in psychiatric consultation or on how to present and discuss the relevant options [16–21]. Existing studies suggest that the use of a shared decision making (SDM) process in psychiatric medication consultations is preferred for making mental health care decisions characterized by uncertainty and when more than one reasonable option is available [22]. Indeed, a recent review by the Australian National Mental Health Consumer Carer Forum on the topic of psychiatric medications concluded, among other things, that a SDM approach encourages the empowered use of psychiatric medications as part of the recovery process and positions patients as active agents in their own recovery process [23].

SDM is a health communication model that helps patients and clinicians make treatment decisions jointly. It gives both parties a framework and legitimacy for the discussion [24], focuses on the patient–clinician interaction, and encourages open dialogue in which both parties have expertise, which should lead to the choice of a consensus plan [25–27]. From an ethical perspective, SDM embraces patients’ experiences, autonomy, and empowerment, which are aligned with personal recovery values and self-determination [28, 29].

One promising approach to operationalizing SDM in the medical consultation is the use of an encounter decision aid (EDA) to help patients and clinicians discuss relevant treatment options, support patients in exploring what is clinically available, and incorporate patient preferences [30, 31]. EDAs are evidence-based tools used before, during, or after a medical encounter to supplement the clinical consultation and to help patients make an informed, deliberate choice among treatment options [32, 33]. EDAs differ from health education materials because they make explicit a specific decision [34], and simplify the information about the patient’s condition and his or her options for treatment, screening, or diagnostic procedures. Previous research on the effects of EDA on various patient populations generally supports the potential positive effects of EDAs on patients’ medical decision-making: improving their knowledge regarding their treatment options and risk perceptions, reducing their level of decisional conflict, and decreasing the proportion of people who remain undecided [31]. However, this review included only two studies of EDAs in mental health [16, 35], both showing the effects of the EDA on uptake of psychoeducation [16] or completion of psychotherapy [35], not on antipsychotic medications adherence, which is strongly different from participation in psychotherapy or psychoeducation. Therefore, although Stacey et al. [31] found strong evidence when the patient choice is about whether to undergo surgery or treatment for heart failure or diabetes, their conclusions cannot be easily extended to patients with mental disorders.

Indeed, previous reviews focusing specifically on SDM in mental health have recommended using decision aids when discussing treatment options [17, 36], but similar biases and methodological limitations in the included articles, as with the findings by Stacey et al. [31], still exist. A recent review of SDM interventions in mental health by Zisman-Ilani et al. [37] included, in addition to randomized control trials (RCT), non-RCT studies and conceptual articles. The authors included 31 articles, of which 12 have described decision aids (or EDAs); only seven (of these 12 articles) were research articles and described an evaluation of a decision aid. Results were mixed; in three studies that used a decision aid, without supplemental elements before or after introducing it (such as goal setting), there was at least one significant outcome related to improved health behaviors (i.e. adherence, service utilization) [19, 38], mental health symptoms [39], and SDM-related outcomes (i.e. involvement, knowledge, self-efficacy). One study [40] reported no significant effect of the decision aid on patient outcomes. Four additional studies, that used a decision aid with supplemental elements, were found to have at least one positive significant effect on SDM-related outcomes (i.e. involvement, knowledge, self-efficacy) [16, 18, 41], and one study showed improved engagement in preferred treatment (psychotherapy, not medications) [35].

To help address the concerns from the limited studies available from these reviews, our purpose in this study was to develop a tool to help patients with psychosis who have stabilized, their carers, and clinicians explicitly address the daily dilemma of continuing, adjusting, or discontinuing antipsychotics during the mental health consultation. Specifically, our aims were: (1) to develop an EDA for antipsychotic medications decision-making that formally addresses the three options (continue, adjust, or stop); and, (2) to revise the tool based on a qualitative user-testing study with potential users that was designed to evaluate the clarity, usability, and potential barriers to and facilitators of future implementation of the new EDA in psychiatric consultations.

## Methods

The EDA was developed according to the recommendations of the International Patient Decision Aid Standards (IPDAS) collaboration [42], the IPDAS instrument (IPDASi) [43], and the previous experience of developing 50 Option Grids™ decision aids. We used a community-based participatory research approach both in developing the EDA and during the evaluation of the proposed EDA by clinicians, carers and consumers [44]. The study had two phases: (1) EDA development, and (2) user testing.

### Encounter decision aid development

We chose a specific format for the EDA, the Option Grid decision aid for clinical encounters, that is short and that can be implemented in routine care during medical encounters [45]. This format provides a simple one-page table with rows containing frequently asked questions by patients about their treatment options and the benefits, risks, and implications of differing decisions. The columns display the treatment options available for the health care decision in question: here, continuing, adjusting, or discontinuing antipsychotic medications.

Creating an Option Grid decision aid of this nature requires a process that brings together the most current scientific evidence and filters that data through a team of health care experts. This team, which most often consists of service users, clinicians, and researchers, rigorously synthesizes evidence from systematic reviews and the most reputable clinical guidelines in an editorial process, hence, the name “editorial research team”.

Our multidisciplinary editorial research team included people with lived experience of serious mental illness, psychiatrists, psychologists, a pharmacist, and decision scientists. The team developed the EDA between March 2015 and March 2016 within a larger project to create technical assistance materials for use in first-episode-psychosis programs. As an editorial research team, we were not only involved in the development and user-testing of the tool but also highly involved in the process of summarizing the evidence in an editorial process, especially when we had to translate from primary or secondary research studies to words and sentences that are accessible to patients and other end-users of the tool. Our team met regularly to discuss development of the EDA and the user-testing strategy and employed an iterative process [46] to create a final version for the study.

The team focused on the often-neglected decision node of what to do with antipsychotic medication management after a person has experienced symptom relief. After opting to focus on medication decisions after initial stabilization, the team identified a series of frequently asked questions that can typify an informed clinical interaction following stabilization from the first episode. The questions that were ultimately selected are portrayed in the rows of the EDA and address various aspects of the three decisions that service users can make following stabilization: Continuing on the medication regime that was used in their initial treatment; Adjusting their antipsychotic medications and/or adding medication for side effects; or Stopping the medications. Following the identification of these questions, the research editorial team reviewed research literature to determine the most accurate responses to the questions. Systematic reviews, treatment guidelines, meta-analyses, and individual studies were included. Based on this process, draft answers to each of the frequently asked questions were reviewed by the team for accuracy and clarity. Each cell in the EDA matrix is linked to an evidence document (Additional file 1) that references the research literature that supports the answers. When no research base is available for a question, the opinion of the editorial research team is cited.

### User-testing study

Once an Option Grid decision aid is constructed, it is put through a series of user tests and is refined until a final version is agreed upon. This document is then used during clinical encounters as a scaffolding of information that prompts a deeper conversation about individuals’ specific desires for particular health care decisions. Ultimately, an Option Grid is meant not to simply be a stand-alone summary but a tool used to promote collaboration and deliberation.

Our user-testing included individual semistructured interviews with people from six stakeholder groups: persons with first and long-term psychosis, family members of patients, psychiatrists, mental health counselors, and administrative staff at mental health clinics. Our team (YZI, DS, MM, GE) developed an interview guide with three broad sections: (1) general impression of the EDA and its purpose; (2) content, wording, accuracy, and visual appearance; and (3) perspectives on usability and implementation in psychiatric settings (Table 1). We adapted the interview guide to fit the six groups of interviewees.

**Table 1:** The semistructured interview guide for patients

General impression and purpose:	
• Can you describe in your own words the purpose of the grid?	
• What is your overall impression of the grid?	
• In your opinion, who is the grid intended for?	
• How helpful do you think such a tool would be for you for decisions about the use of antipsychotic medication?	
Content, wording, and appearance:	
• Is there anything you’d add, delete, or change about the opening instructions of the DA?	
• Is there anything you’d add, delete, or change about the information in the row ‘What does this involve?’ †	
• Are there other questions that you feel would be important to include?	
Usability and potential contribution to the psychiatric consultation:	
• In your opinion, how would this tool best be incorporated into aclinic’s workflow? (prompts: Before the visit at home? Before visit by clinic staff? During visit? After visit?)	
• Do you think there is room for such a tool in psychiatric care?	
• Would you prefer a static version (paper and pencil) or an interactive paper (for example on a tablet or mobile device)?	
• Would you like the clinician to use this tool as part of your meeting/consultation? (Why?)	
• How do you think patients will respond to such a tool?	
• How do you think clinicians will respond to such a tool?	
†Four more questions like this were asked for each of the additional rows in the decision aid.	

### Recruitment and procedures

Our team advertised the study and recruited potential interviewees with the help of colleagues at two community mental health clinics in rural New Hampshire (patients with long-term illness, psychiatrists, mental health counselors, and administrators), a mental health clinic for young adults with first-episode psychosis in the New York City area affiliated with Mental Health America (patients with first-episode psychosis), and a support group for family members run by the National Alliance on Mental Illness (family members of people with a history of psychosis). We chose these sites based on the relationships between some members of the editorial research team and these settings; some members of the team helped open doors and access the specific clinics and sites. However, these members had no direct interaction with the study participants. In addition, the lead author (YZI) conducted all interviews and had no prior relationships with the clinics or the participants.

Using non-probability purposive sampling, we recruited a convenience sample of interviewees based on population characteristics and on the study’s objective to achieve a final sample of diverse participant groups [47–49]. Specifically, we used expert sampling because our research requires first-hand experts in relation to antipsychotics (prescribing, consulting, using, and caring for users) to capture knowledge rooted in a particular form of expertise. It is common to use this purposive sampling technique in the early stages of a research process, when the researchers are seeking to become better informed about the topic at hand before embarking on a study. Eligibility criteria varied by group category. Patients (first-episode psychosis and long-term illness) had experienced at least one episode of psychosis for which they took antipsychotic medications, were age 18 years or older, and had a chart diagnosis of schizophrenia, schizoaffective disorder, schizophreniform disorder, delusional disorder, or psychosis not otherwise specified. Interviewees were excluded if they could not speak, write, or read English or could not participate in an interview because their symptoms of psychosis were not stabilized by the time of the interview. Patient interviews were conducted face-to-face. Interviews with family members, clinicians, and administrative staff were conducted face-to-face or by telephone.

We obtained verbal consent from all respondents at the time of the interviews and written consent for face-to-face interviews. All interviewees were assured that participation was voluntary and that all information collected would be confidential and used for research purposes only. Interviewees were offered a $20 honorarium for their participation after the face-to-face interviews or $10 for telephone interviews (via mail). Each interview lasted about 30 min. The Committee for the Protection of Human Subjects at Dartmouth College approved the study.

### Analysis

We used an iterative coding and analysis process with constant comparison [50, 51]. Two authors (YZI, KG) coded the interviews to ensure intercoder reliability and conferred weekly [52]. Coders resolved discrepancies through discussion. For example, codes were combined or split after conferring with the other coder. After we reached agreement on the themes, YZI wrote an evolving qualitative memo, which was reviewed by KG biweekly. Other authors (GE and DS) commented on ongoing drafts of the results in both content and presentation. These versions of the tables and figures were presented to the entire editorial research team to gather their feedback.

## Results

### The encounter decision aid for antipsychotic medications

An EDA targeting three options (continue, adjust, or stop medication) was developed (Additional file 2).

### User testing of the encounter decision aid

We recruited 28 potential participants but excluded four: one could not read**,** another was psychotic at the beginning of the interview and could not participate in the interview, and two were under the age of 18. The final sample included 24 interviewees: five patients with first-episode psychosis, six persons with long-term illness, five family members of persons with psychosis (not related to the interviewed patients), six clinicians (three psychiatrists and three mental health counselors), and two health care administrators. All patients in the study had an initial episode of psychosis or a long-term illness but had stabilized. The sample size was sufficient to ensure diversity of perspectives for the purpose of our study [53] and matched the development process of 50 existing EDAs at Dartmouth College. Our interviewees represent diverse ages and genders. Their ages ranged from 19 to 70 years, with family members 10 years older on average than other interviewees. About half the interviewees were women (*n* = 14, 58.3%). All patients had used or were currently using antipsychotic medication.

### Domain 1: general impression and purpose of the decision aid

#### First impression: purpose and target audience

All participants clearly identified the EDA’s purpose as a tool to help in decision-making regarding antipsychotic medications. They also almost uniformly reported that the EDA was intended for people with a history of psychosis. When prodded by the interviewer about the EDA’s utility to clinicians, most interviewees added that it might help clinicians. For example, one patient with a first-episode psychosis suggested that the EDA might help clinicians accustomed to making treatment decisions with little input from their patients become more aware of patients’ perspective and desires: “I think it could help [clinicians] too because they’re kind of old school [.. . and. ..] [t]hey’re more on the side of you either taking it or switching medication.” Only one psychiatrist thought the EDA was primarily for clinicians.

A patient with a long-term illness noted that the EDA might be more helpful to newly diagnosed people. By contrast, a clinician commented that the EDA might be most useful to people who have “reached a level of either stability or. .. they feel they’re doing very, very well” and begin to question their medication’s utility. A psychiatrist noted that uses of the EDA could extend to any psychiatric encounters because “most people do stop their antipsychotic medication or skip doses. .. [E]ven when you start, it’s unrealistic to think that someone is always taking their medication.”

#### First impression: utility

A few patients told us that the EDA would be useful to them. One patient said, “I would really like to be able to use [the EDA], because it would help me focus on what I need to pay attention to in case something either went good or not so good when we try changing my medication.” Psychiatrists and counselors commented that the EDA may enhance discussions about risks and benefits of antipsychotic medications. They cautioned that focusing on side effects of antipsychotics may push their patients to consider stopping their use: “I’d feel great if [patients] actually thought it out, instead of just deciding [to stop medications]. .. without thinking about, okay, well then what might happen?”

#### First impressions: family concerns

Of all stakeholders, family members were the most guarded in their first reactions to the EDA. Some found unthinkable the idea of discussing the option of stopping medication. One family member thought the EDA might help someone with a high level of reading comprehension who can make rational decisions: “I think it’s intended for somebody who can read and make judgments and understand it. .. Their decision-making may not be at a level where they can make a competent decision.” Another family member was more ambivalent about the idea of involving patients in treatment decision-making: “I wonder how someone who has severe mental illness and is controlling it through medication and also through counseling would be able to judge this on their own without the help of the professionals [.. .] how do you even bring. .. up [stopping/adjusting medications] as a topic? Because in some families, that’s like lighting a. .. powder keg. And other families, I think it’s a great relief that somebody does finally bring it up.”

#### Domain 2: suggested changes to the content, wording, and appearance of the decision aid

**Fig. 1:**
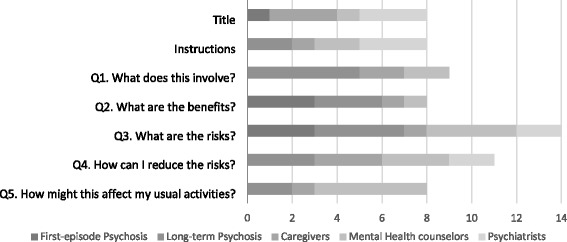
Number of quotations† regarding the appearance and content of the EDA

†Suggested changes/dissatisfaction with the content. Administrators’ responses are missing, as they were not asked about the specific content of the EDA.

Most interviewees liked the EDA’s content, wording, and appearance. Figure 1 summarizes the suggested revisions to the EDA’s content, by question and by stakeholder group. A total of 66 revisions were offered (we did not count agreement with the text). Questions 3 and 4 (see below) elicited the highest rates of dissatisfaction with the content. Patients with long-term illness and mental health counselors suggested the most revisions to the EDA’s content.

##### Suggested revisions to the title and instructions

Table 2 describes the user-testing version of the EDA, dated March 2016, and the final version, dated July 2016. Interviewees suggested using a simple, short, descriptive title to reach a wide audience*.* One psychiatrist noted that the term *psychosis* may be confusing to some patients, particularly if they think of themselves as taking the medication “for an episode of confusion or an episode of extreme agitation and disorientation.” One family member suggested that the EDA should frame the consultation as a decision-making meeting from the beginning, to facilitate the process. This interviewee suggested using relevant buzzwords in the instructions to reflect that the EDA is intended to facilitate engagement in the treatment process and to provide support in medication decision making. The final wording of the instructions is as follow: “Use this decision aid to help you, your caregiver, and your doctor (prescriber) decide how to best manage your medication. This decision aid is most appropriate for people who take medication for psychosis and for those who have had psychosis for the first time.”

**Table 2:** Changes in the encounter decision aid based on participants’ feedback from the user testing to the final version

		Continue	Adjust	Stop
Title	March 2016	Use of antipsychotic medications after recovering from past episode of psychosis		
	July 2016	Antipsychotic medication: continue, adjust, or stop? for people who have stabilized		
Instructions	March 2016	Use this Option Grid™ decision aid to help you and your clinician consider how best to manage your antipsychotic medications.		
	July 2016	Use this decision aid to help you, your caregiver, and your doctor (prescriber) decide how to best manage your medication. This decision aid is most appropriate for people who take medication for psychosis and for those who have had psychosis for the first time.		
Q1	March 2016	Making no changes to the medications you take.	Taking more or less medication, adding or changing if needed. It is best to take the fewest number of medicines at the lowest dose. This often needs adjustments and is best done with help from your clinician.	Gradually stopping your medications. This is best done with help from your clinician and may involve learning other strategies to manage your symptoms.
	July 2016	Making no changes to your medications. Please ask your doctor about the effects of continuing to use antipsychotic medications. It is important to work closely with your doctor.	Taking more or less medication, adding or changing if needed. It is best to take the fewest number of medications at the lowest dose that works. Adjusting medication is best done with help from your doctor.	Slowly stopping your medications. This is best done with help from your doctor and may involve learning other ways to manage your symptoms.
Q2	March 2016	You can expect things to stay the same, both the good and the bad.	You can experience fewer medication related side effects, such as sleepiness, uncontrollable movements and weight gain.	You can avoid medication side effects such as sleepiness, uncontrollable movements and weight gain.
	July 2016	You can expect things to stay the same. Medications can help you focus less on symptoms and more on the things that are important to you, like work or school.	You may have fewer medication side effects, such as sleepiness, uncontrollable movements and weight gain.	No revisions
Q3	March 2016	You might be on too high dose or too much medication. Common side effects are sleepiness, uncontrollable movements and weight gain.	Problems may come back on a lower dose, and you may have more difficulty working or concentrating. You may need more check ups to be sure that symptoms don’t come back.	Symptoms may get worse if medications are stopped. Roughly 80 in every 100 people (80%) will suffer symptoms again in one year after stopping medications. These symptoms may cause you to go back into the hospital.
	July 2016	Common side effects are sleepiness, uncontrollable movements, weight gain, and other effects such as sexual problems. You might not be on the dose or combination of medications that’s best for you.	Symptoms may come back on a lower dose, and you may find it hard to work or concentrate. You may need more check-ups with your doctor to make sure that symptoms don’t come back.	Your symptoms may get worse if you stop taking medication. Roughly 80 in every 100 people (80%) will have symptoms again in the year after stopping medications. These symptoms may cause you to go back to a hospital or psychiatric community clinic.
Q4	March 2016	Try to find ways to limit side effects. You might want to eat health food, join patient groups and stay physically active. Avoid using alcohol or street drugs because they can make it hard to understand if your medications are working	Keep track of how you feel, using a journal so that you know if your medications are helping or not, and share it with your clinician. Maybe ask someone to give you feedback about how you are doing. Avoid using alcohol or street drugs because they can make it hard to understand if your medications are working.	Make sure that you have frequent contact with your clinician. Follow the schedule as you reduce the the medicine dose. Don’t downplay your symptoms if they come back. Avoid using alcohol or drugs because they can make it hard to understand if your medications are working.
	July 2016	You can try to find ways to limit side effects, such as by eating healthy food, joining patient groups and staying physically active. Avoid alcohol and street drugs.	Keep track of how you feel and talk with your doctor. You can try support groups, mobile apps, or writing in a journal so that you know if your medications are helping or not. You can ask someone to give you feedback about how you are doing. Avoid alcohol and street drugs.	Talk regularly with your doctor, a mental health counselor, family members or a friend. Follow the schedule as you lower the medication dose. Don’tdownplay your symptoms if they come back. Avoid alcohol and street drugs.
Q5	March 2016	If the medications are being helpful, then you would get back to your usual activities.	Taking the lowest effective dose will cause fewer side effects. Being more alert and less bothered by side effects may help you get back to your normal activities.	It is likely that your symptoms will come back and make it more difficult to do your normal activities. Stay in close contact with your clinician.
	July 2016	No revisions	Taking the lowest dose that works will cause fewer side effects. This may help you get back to your usual activities.	Once your symptoms improve, you will be able to go back to your usual activities. However, your symptoms will likely come back at some point. Stay in touch with your doctor.

##### Suggested changes to question 1: what does this involve?

Based on comments by several patients with long-term illness, we changed the information in each of the cells to be less deterministic (Table 2)*.* We also wanted to emphasize that stopping antipsychotics requires a gradual process.

##### Suggested changes to question 2: what are the benefits?

Participants identified an imbalance between the three options and a bias toward stopping antipsychotic medications. One patient with a first-episode psychosis commented: “I think you should have more of the benefits [of the medications] because it really does help [.. .] You can just tell right away.”

##### Suggested changes to question 3: what are the risks?

As with Question 2, one patient mentioned that there should be more balance between each option. A patient with a long-term illness noted that sexual problems are a major side effect that should be specifically mentioned. Interestingly, a patient with a first-episode psychosis shared with us that the need for frequent check-ups with the community psychiatrist when adjusting or stopping antipsychotics is an important consideration for this decision. She felt that young people might prefer to adhere to their suggested dose in order to avoid frequent contact with the psychiatrist, whereas patients with long-term psychosis may be more likely to reduce and taper their medications.

##### Suggested changes to question 4: how can i reduce the risks?

Patients mentioned that having a trusting relationship with people other than their clinician can be valuable because these individuals can provide feedback about the patient’s mental health. Patients mentioned additional strategies for illness management such as participation in support groups and use of mobile apps.

##### Suggested changes to question 5: how might this affect my usual activity?

Interviewees suggested ending the EDA with a positive take-home message, using the active voice, to instill/foster hope. A patient with a long-term illness explained that some symptoms are tolerable and that eliminating them is not always the ultimate goal.

#### Domain 3: Usability and potential contribution to the psychiatric consultation

Interviewees felt that the EDA can be useful and feasible in routine care. An administrator said, “[The EDA] gives them something to follow along with as [the patient, family member, or clinician is] talking.” This view was supported by several patients. However, some interviewees felt that the usability would depend on the patient’s stage of illness:

I think [patients] would respond well [to the EDA] depending on where they’re...in their disease. If it’s not immediate crisis and they’re able to contemplate these sort of things, then I think it would be very helpful for them. (Family member)

Some interviewees felt that the EDA may facilitate better involvement in mental healthcare:

It gives you a choice [...] you’re able to look at what are the benefits, side effects and stuff, how continuing medication or stopping the medication or adjusting the medication, it gives you a choice to look at all three of them. (Patient with long-term psychosis)

Family members mentioned that the EDA may increase the patient’s awareness of their options including stopping medication, and that this option/issue is not usually discussed: “I think a lot of people wouldn’t have even considered these options [unless their provider shows them the EDA].”

## Discussion

The newly developed EDA was positively evaluated by the different stakeholders who participated in user-testing. The EDA was perceived as usable, context-appropriate, and potentially feasible in psychiatric consultations. Some respondents, particularly patients and family members, were concerned that the early version of the EDA strongly advocated against antipsychotic medications, which led to revisions to the final version. Interviewees encouraged us to present the information neutrally. This recommendation is aligned with previous work on the role of persuasive approaches in medical decision-making and the need to determine whether medical decisions are the result of a doctor–patient partnership or of persuasive tactics based. on power and hierarchical relationships [54]. Persuasive theories are one subset of health communication theories; they can be applied at many levels including intrapersonal, interpersonal, organizational, and mass communication [55]. Accordingly, persuasion can play a critical role in doctor–patient communication, as it is often the intent of a practitioner to shape, reinforce, or change a patient’s behavior [56], especially when discussing medication and treatment adherence [55], and even more in psychiatric settings, where patients are often questioned about their ability and capability to make treatment decisions [17, 57].

Our respondents raised the issue of patients’ ability to make decisions. This is particularly relevant to patients with schizophrenia spectrum disorders and serious mental illness, who may lack awareness of their illness [58–60]. However, findings from the last decade about SDM in mental health provide evidence that people with mental conditions, including schizophrenia [16], can effectively participate in SDM with their health provider [37] and will make medication decisions independently without clinical guidance [23]. Therefore, because people with long-term illness provided most of the revisions to the EDA’s wording and content, this may demonstrate their ability and capacity to engage in these ideas as well as anyone else, challenging perhaps the assumption that they lack capacity.

Family members were guarded in their initial reactions to the EDA, perhaps fearing that discussion of stopping medication might suggest an alternative that would harm their family member. Another explanation might be related to a sense of family burden in caring for a loved one with a mental illness [61–63], and a fear of a sudden increase in this burden may explain family members’ concerns about the EDA. Because family members play a major role in a triadic decision-making in mental health care [64, 65], their concerns are valid and should be addressed to facilitate and support the discussion on medication management. The present EDA and future SDM tools should include family members as targeted end-users, along with patients and clinicians, to support implementation and increase buy-in.

Our study has several strengths: First, we presented a heterogeneous overall sample representing unique access to the different stakeholder groups that created an important diversity of participant groups in the final sample. Second, we have described the development of a tool using a participatory approach that considered participants “active consultants” rather than “research subjects.” This research approach allows people with severe mental illness / psychotic illness, who may be largely excluded from decisions about their treatment, to become actively involved in the research and provide important feedback. Therefore, not only may SDM and tools such as decision aids help shift the power balance in medical encounters to help address coercive treatment, but also the participatory approach may help patients feel valued for their input, which, in turn, may further empower them. These strengths allowed us to explore and compare the different perceptions and perspectives of people with lived experiences of antipsychotic medication management. Yet, several limitations should be noted: First, the relatively small sample in each group may affect the generalizability of the results. Second, the intention of this article is not to venture into making suggestions about clinical impact, as this was a small, qualitative user-testing. Future field-testing research in various settings, such as clinics, hospitals, and community programs, is needed. In addition, future potential research would require a further study with much larger samples to test the effectiveness of the EDA and should include clinical and more psychosocial impact measurements.

## Conclusions

Our participants, people who are involved in antipsychotic medication management, found the EDA to be valuable and acceptable. Our aim in this research and article was not to provide answers or to solve the dilemma of whether to continue, adjust, or stop antipsychotic medications. We believe it is a very personal decision that has no “one-size-fits-all” answer. Our aim in this project was to give voice and room for such a question/dilemma to be openly addressed as part of the psychiatric encounter and to help patients and other people involved in their care to “lay their cards on the table” and to openly raise concerns and questions about antipsychotics. As one of our interviewees shared with us: “I think a lot of people wouldn’t have even considered these options [unless their provider shows them the EDA].” We hope that this EDA will help patients and carers openly raise the dilemma, without fearing the response to and stigma associated with people with serious mental illness who wish to stop or adjust their medications [4, 66]. We anticipate that giving this dilemma room and space to be addressed as part of a psychiatric consultation may have a personal-rehabilitative benefit for the person with the illness as well as for others involved in his/her care. We hope that use of this EDA in psychiatric consultations will contribute to making personalized-better choices, ones that patients understand, agree to, and are more likely to follow, and that use of the EDA will encourage an open discussion between patients and their care team, improving engagement in psychiatric care.

## Additional files

Additional file 1: An evidence document that references the research literature that supports the answers. (PDF 63 kb)

Additional file 2: Encounter Decision Aid (EDA). (DOCX 15 kb)

## Abbreviations

EDA/s: Encounter decision aid/s
SDM: Shared decision making
