# Supplementary material for: Continue, adjust, or stop antipsychotic medication: developing and user testing an encounter decision aid for people with first-episode and long-term psychosis
Source: BMC Psychiatry. 2018 May 22;18:142. doi: 10.1186/s12888-018-1707-x (PMC5963160; doi:10.1186/s12888-018-1707-x)
Supplement: Supplementary file 1 — An evidence document that references the research literature that supports the answers. (PDF 63 kb) [file 12888_2018_1707_MOESM1_ESM.pdf]

Antipsychotic medication: continue, adjust, or stop?

Evidence document

What does this involve?

Continue antipsychotic medications

McEvoy, J.P., Lieberman, J.A., Perkins, D.O. et al. Efficacy and tolerability of olanzapine, quetiapine and risperidone in the treatment of early psychosis: A randomized, double blind 52 week comparison. American Journal of Psychiatry. 164:1050-1060, 2007.

Schooler, N.R. The efficacy and safety of conventional and atypical antipsychotics in first-episode schizophrenia: A review of the literature. Clinical Schizophrenia and Related Psychoses. April, 2007.

Kahn, R.S., Fleischhacker, W.W. & Boter, H. Effectiveness of antipsychotic drugs in first-episode schizophrenia and schizophreniform disorder: An open randomized clinical trial. The Lancet, 371:1085-1097, 2008.

Noordsy, D.L., Torrey, W.C., Mead, S. et al Recovery-oriented psychopharmacology: Redefining the goals of antipsychotic treatment. Journal of Clinical Psychiatry. 61(Suppl 3): 22-28, 2000.

Editorial consensus.

Adjust antipsychotic medications

Essock, S.M., Covell, N.H., Davis, S.M,, et al Effectiveness of switching antipsychotic medications. American Journal of Psychiatry. 163(12):2090-2095, 2006.

Mukundan, A., G., Faulkner, T., Cohn, & G. Remington. Can changing antipsychotic medication improve side effects like increases in weight, blood sugar and cholesterol? Cochrane Review, 2008

Uchida, H., Suzuki, T., Takeuchi, H., Arenovich, T. & Mamo, D. Low dose vs standard dose of antipsychotics for relapse prevention in schizophrenia: Meta-analysis. Schizophrenia Bulletin, 37(4):788-799, 2009.

Buchanan, R., Kreyenbuhl, J. et al The 2009 schizophrenia PORT psychopharmacological treatment recommendations and summary statements. Schizophrenia Bulletin. 36(1):71-93, 2010.

McGorry, P., Alvarez-Jimenez, M. & Killackey, E. Antipsychotic medication during the critical period following remission from first-episode psychosis. Journal of the American Medical Association – Psychiatry, 70(9):898-900, 2013.

Robinson, D.G., Schooler, N.R. & Majnu, J. Prescription practices in the treatment of first episode schizophrenia spectrum disorders: Data from the national RAISE-ETP study. American Journal of Psychiatry, 172:237-248, 2015.

Editors: David Shern, Glyn Elwyn, Julie Kreyenbuhl, Robert Drake, Patricia Deegan, Lisa Dixon, Anthony Lehman, William Torrey, Manish Mishra, Yaara Zisman-Ilani
ISBN: 978-1-941487-17-4
License: CC BY-NC-ND 4.0 (International)
More information: http://optiongrid.org

Dixon L., & Stroup, T. Medications for first episode psychosis: Making a good start. American Journal of Psychiatry, 172(3):209-211, 2015.

Cerovecki, A., Musil, R,, Klimke, A. et al. Withdrawal symptoms and rebound syndromes associated with switching and discontinuing atypical antipsychotics: Tfheoretical background and practical recommendations. CNS Drugs, 27(7):545-572, 2013.

Moore, T.A., Buchanan, R.W. & Buckley, P.F. The Texas medication algorithm project Antipsychotic algorithm for schizophrenia: 2006 update. Journal of Clinical Psychiatry, 68(11):1751-1762, 2006.

Fraser, R., Berger, G., Killackey, E. & McGorry, P. Emerging psychosis in young people – part 3. Australian Family Physician, 35(5):329-332, 2006.

Stop antipsychotic medications

Hogan, D. Strategies for discontinuing psychotropic medications. Canadian Geriatric Society Journal of CME, 4(2):14-18, 2014.

Harrow, M., Jobe, T.H., & Faull, R.N. Do all schizophrenia patients need antipsychotic treatment continuously throughout their lifetime? A 20 year longitudinal study. Psychological Medicine, 42: 2145-2155, 2012.

Fraser, R., Berger, G., Killackey, E. & McGorry, P. Emerging psychosis in young people – part 3. Australian Family Physician, 35(5): 329-332, 2006.

Saloman, C. & Hamilton, B. Antispychotic discontinuation syndromes: A narrative review of the evidence and its integration into Australian mental health nursing textbooks. International Journal of Mental Health Nursing, 23:69-78, 2014.

Muesser, K.T., Corrigan, P.W., Hilton, D.W. et al. Illness management and recovery: A review of the research. Psychiatric Services, 53(10):1272-1284, 2002

What are the benefits?

Continue antipsychotic medications

Leucht, S., Tardy, M., Komossa, K., et al Maintenance treatment with antipsychotic drugs for schizophrenia. The Cochrane Library (2012). http://www.cochrane.org/CD0080...

Karson, C., Duffy, R.A., Eramo, A., Nylander, A.G. & Offord, S.J. Long-term outcomes of antipsychotic treatment in patients with first-episode schizophrenia: A systematic review. Neuropsychiatric Disease and Treatment, 12:57-67, 2016.

Mizrahi, R., Bagby, R.M., Zipursky, R.B. and Kapur, S. How antipsychotics work: The patients perspective. Neuro-Psychopharmacology & Biological Psychiatry, 29, 859-864, 2005.

Editorial consensus.

Editors: David Shern, Glyn Elwyn, Julie Kreyenbuhl, Robert Drake, Patricia Deegan, Lisa Dixon, Anthony Lehman, William Torrey, Manish Mishra, Yaara Zisman-Ilani
ISBN: 978-1-941487-17-4
License: CC BY-NC-ND 4.0 (International)
More information: http://optiongrid.org

Adjust antipsychotic medications

McEvoy, J.P., Lieberman, J.A., Perkins, D.O. et al Efficacy and tolerability of olanzapine, quetiapine and risperidone in the treatment of early psychosis: A randomized, double blind 52 week comparison. American Journal of Psychiatry, 164:1050-1060, 2007.

Viguera, A., Baldessarini, R., Hegarty, J., van Kammen, D. & Tohen, M. Clinical risk following abrupt and gradual withdrawal of maintenance neuroleptic treatment. Archives of General Psychiatry, 54(1):49-55, 1997.

Schooler, N.R. The efficacy and safety of conventional and atypical antipsychotics in first-episode schizophrenia: A review of the literature. Clinical Schizophrenia and Related Psychoses, 1(1):27-42, 2007.

Leucht, S., Tardy, M., Komossa, K. et al. Antipsychotic drugs versus placebo for relapse prevention in schizophrenia: A systematic review and meta-analysis, The Lancet, 379:2063-2071, 2012.

Buchanan, R., Kreyenbuhl, J. et al. The 2009 schizophrenia PORT psychopharmacological treatment recommendations and summary statements. Schizophrenia Bulletin 36(1):71-93 2010.

Bruijnzeel, D., Suryadevara, U. & Tandon, R. Antipsychotic treatment of schizophrenia: An update. Asian Journal of Psychiatry, 11:3-7, 2014.

Stop antipsychotic medications

McEvoy, J.P., Lieberman, J.A., Perkins, D.O. et al Efficacy and tolerability of olanzapine, quetiapine and risperidone in the treatment of early psychosis: A randomized, double blind 52 week comparison. American Journal of Psychiatry, 164, 1050-1060, 2007.

Viguera, A., Baldessarini, R., Hegarty, J., van Kammen, D. & Tohen, M. Clinical risk following abrupt and gradual withdrawal of maintenance neuroleptic treatment. Archives of General Psychiatry, 54(1):49-55, 1997.

Schooler, N.R. The efficacy and safety of conventional and atypical antipsychotics in first-episode schizophrenia: A review of the literature. Clinical Schizophrenia and Related Psychoses, 1(1):27-42, 2007.

Leucht, S., Tardy, M., Komossa, K. et al. Antipsychotic drugs versus placebo for relapse prevention in schizophrenia: A systematic review and meta-analysis, The Lancet, 379: 2063-2071, 2012.

Buchanan, R., Kreyenbuhl, J. et al The 2009 schizophrenia PORT psychopharmacological treatment recommendations and summary statements. Schizophrenia Bulletin 36(1):71-93 2010.

Bruijnzeel, D., Suryadevara, U. & Tandon, R. Antipsychotic treatment of schizophrenia: An update. Asian Journal of Psychiatry, 11:3-7, 2014.

What are the risks?

Editors: David Shern, Glyn Elwyn, Julie Kreyenbuhl, Robert Drake, Patricia Deegan, Lisa Dixon, Anthony Lehman, William Torrey, Manish Mishra, Yaara Zisman-Ilani
ISBN: 978-1-941487-17-4
License: CC BY-NC-ND 4.0 (International)
More information: http://optiongrid.org

Continue antipsychotic medications

McEvoy, J.P., Lieberman, J.A., Perkins, D.O. et al Efficacy and tolerability of olanzapine, quetiapine and risperidone in the treatment of early psychosis: A randomized, double blind 52 week comparison. American Journal of Psychiatry, 164:1050-1060, 2007.

Viguera, A., Baldessarini, R., Hegarty, J., van Kammen, D. & Tohen, M. Clinical risk following abrupt and gradual withdrawal of maintenance neuroleptic treatment. Archives of General Psychiatry, 54(1):49-55, 1997.

Schooler, N.R. The efficacy and safety of conventional and atypical antipsychotics in first-episode schizophrenia: A review of the literature. Clinical Schizophrenia and Related Psychoses, 1(1):27-42, 2007

Leucht, S., Tardy, M.,Komossa, K., Heres, S., Kissling, W., Salanti, G. et al. Antipsychotic drugs versus placebo for relapse prevention in schizophrenia: A systematic review and meta-analysis, The Lancet, 379:2063-2071,2012.

Buchanan, R., Kreyenbuhl, J. et al The 2009 schizophrenia PORT psychopharmacological treatment recommendations and summary statements. Schizophrenia Bulletin. 36(1):71-93, 2010.

Bruijnzeel, D., Suryadevara, U. & Tandon, R. Antipsychotic treatment of schizophrenia: An update. Asian Journal of Psychiatry, 11:3-7, 2014.

Smith, S., O'keane, V., & Murray, R.. Sexual dysfunction in patients taking conventional antipsychotic medication. The British Journal of Psychiatry, 181(1):49-55, 2002.

Montejo, Á. L., Majadas, S., Rico‐Villademoros, F. et al Frequency of sexual dysfunction in patients with a psychotic disorder receiving antipsychotics. The Journal of Sexual Medicine, 7(10): 3404-3413, 2010.

Robinson, D.G., Schooler, N.R. & Majnu, J. Prescription practices in the treatment of first episode schizophrenia spectrum disorders: Data from the national RAISE-ETP study. American Journal of Psychiatry, 172:237-248, 2015.

Essock, S.M., Covell, N.H., Leckman-Westin, E. et al. Identifying clinically questionable psychotropic prescribing practices for Medicaid recipients in New York state. Psychiatric Services, 60(12):1595-1602, 2009.

Uchida, H., Suzuki, T., Takeuchi, H., Arenovich, T. & Mamo, D. Low dose vs standard dose of antipsychotics for relapse prevention in schizophrenia: Meta-analysis. Schizophrenia Bulletin, 37(4):788-799, 2011

Adjust antipsychotic medications

Viguera, A., Baldessarini, R., Hegarty, J., van Kammen, D. & Tohen, M. Clinical risk following abrupt and gradual withdrawal of maintenance neuroleptic treatment. Archives of General Psychiatry, 54(1):49-55, 1997.

Mukundan, A., G. Faulkner, T. Cohn, & G. Remington. Can changing antipsychotic medication improve side effects like increases in weight, blood sugar and cholesterol? Cochrane Review,

Editors: David Shern, Glyn Elwyn, Julie Kreyenbuhl, Robert Drake, Patricia Deegan, Lisa Dixon, Anthony Lehman, William Torrey, Manish Mishra, Yaara Zisman-Ilani
ISBN: 978-1-941487-17-4

License: CC BY-NC-ND 4.0 (International)

More information: http://optiongrid.org

2008.
http://www.cochrane.org/CD006629/SCHIZ_can-changing-antipsychotic-medication-improve-side-effects-like-increases-in-weight-blood-sugar-and-cholesterol

Kam, S.M., Singh, S.P. & Upthegrove, R. What needs to follow early intervention? Predictors of relapse and functional recovery following first-episode psychosis. Early Intervention in Psychiatry, 9:279-283, 2015.

Karson, C., Duffy, R.A., Eramo, A., Nylander, A.G. & Offord, S.J. Long-term outcomes of antipsychotic treatment in patients with first-episode schizophrenia: A systematic review. Neuropsychiatric Disease and Treatment, 12:57-67, 2016.

Fraser, R., Berger, G., Killackey, E. & McGorry, P. Emerging psychosis in young people – part 3. Australian Family Physician, 35(5):329-332, 2006.

Stop antipsychotic medications

Viguera, A., Baldessarini, R., Hegarty, J., van Kammen, D. & Tohen, M. Clinical risk following abrupt and gradual withdrawal of maintenance neuroleptic treatment. Archives of General Psychiatry, 54(1):49-55, 1997.

Mukundan, A., G. Faulkner, T. Cohn, & G. Remington. Can changing antipsychotic medication improve side effects like increases in weight, blood sugar and cholesterol? Health Cochrane Review, 2008.
http://www.cochrane.org/CD006629/SCHIZ_can-changing-antipsychotic-medication-improve-side-effects-like-increases-in-weight-blood-sugar-and-cholesterol

Zipursky, R., Menezes, N. & Streiner, D. Risk of symptom recurrence with medication discontinuation in first-episode psychosis: A systematic review. Schizophrenia Research,152:408-414, 2014.

How can I lower the risks?

Continue antipsychotic medications

NICE Clinical Guideline CG178: Psychosis and schizophrenia in adults: treatment and management, 2014 - 7.2.8.1

Norman, R.M., Malia, A.K., Manchanda, R. et al Social support and three year symptom and admission outcomes for first episode psychosis. Schizophrenia Research, 80(2-3):227-234, 2005.

Lewis, L. The role of mental health patient organizations in disease management. Practical Disease Management, 9(11):607-617, 2001.

Stone, J.M., Fisher, H.L., Major, B. et al. Cannabis use and first-episode psychosis: Relationship with manic and psychotic symptoms, and with age at presentation. Psychological Medicine, 44(3):499-506 2014.

Faridi, K., Joober, R. & Malla, A. Medication adherence mediates the impact of sustained cannabis use on

Editors: David Shern, Glyn Elwyn, Julie Kreyenbuhl, Robert Drake, Patricia Deegan, Lisa Dixon, Anthony Lehman, William Torrey, Manish Mishra, Yaara Zisman-Ilani
ISBN: 978-1-941487-17-4
License: CC BY-NC-ND 4.0 (International)
More information: http://optiongrid.org

symptom levels in first episode psychosis. Schizophrenia Research, 141:78-82, 2012.

Mueser, K., Gingerich, S. Treatment of co-occurring psychotic and substance use disorders. Social Work Public Health, 28(3-4):424-39. 2013, doi: 10.1080/19371918.2013.774676.

Sheidow, A., McCart, M., Zajac, K. & Davis, M. Prevalence and impact of substance use among emerging adults with serious mental health conditions. Psychiatric Rehabilitation Journal, 35(3):235-43, 2012.

Adjust antipsychotic medications

Noordsy, D.L., Torrey, W.C., Mead, S. et al Recovery-oriented psychopharmacology: Redefining the goals of antipsychotic treatment. Journal of Clinical Psychiatry. 61(Suppl 3):22-28, 2000.

NICE Clinical Guideline CG178: Psychosis and schizophrenia in adults: treatment and management, 2014 8.5.1.2

van der Krieke, L., Emerencia, A. C., Boonstra, N., et al. A web-based tool to support shared decision making for people with a psychotic disorder: randomized controlled trial and process evaluation. Journal of Medical Internet Research, 15(10), e216, 2013.

Mueser K.T. & Gingerich S. Illness self-management programmes. In: Thornicroft, G., Szmukler, G., Mueser K. & Drake R.(eds) Oxford Textbook of Community Mental Health, Oxford: Oxford University Press, 2011.

Stone, J.M., Fisher, H.L., Major, B. et al. Cannabis use and first-episode psychosis: Relationship with manic and psychotic symptoms, and with age at presentation. Psychological Medicine, 44(3):499-506 2014.

Faridi, K., Joober, R. & Malla, A. Medication adherence mediates the impact of sustained cannabis use on symptom levels in first episode psychosis. Schizophrenia Research, 141:78-82, 2012.

Mueser, K., Gingerich, S. Treatment of co-occurring psychotic and substance use disorders. Social Work Public Health, 28(3-4):424-39. 2013, doi: 10.1080/19371918.2013.774676.

Sheidow, A., McCart, M., Zajac, K. & Davis, M. Prevalence and impact of substance use among emerging adults with serious mental health conditions. Psychiatric Rehabilitation Journal, 35(3):235-43, 2012.

Editorial consensus.

Stop antipsychotic medications

Fraser, R., Berger, G., Killackey, E. & McGorry, P. Emerging psychosis in young people – part 3. Australian Family Physician, 35(5), 329-332, 2006.

Saloman, C. & Hamilton, B. Antipsychotic discontinuation syndromes: A narrative review of the evidence and its integration into Australian mental health nursing textbooks. International Journal of Mental Health Nursing, 23:69-78, 2014.

Muesser, K.T., Corrigan, P.W., Hilton, D.W. et al Illness management and recovery: A review of the research.

Editors: David Shern, Glyn Elwyn, Julie Kreyenbuhl, Robert Drake, Patricia Deegan, Lisa Dixon, Anthony Lehman, William Torrey, Manish Mishra, Yaara Zisman-Ilani
ISBN: 978-1-941487-17-4
License: CC BY-NC-ND 4.0 (International)
More information: http://optiongrid.org

Psychiatric Services, 53(10), 1272-1284, 2002.

Stone, J.M., Fisher, H.L., Major, B. et al. Cannabis use and first-episode psychosis: Relationship with manic and psychotic symptoms, and with age at presentation. Psychological Medicine, 44(3):499-506 2014.

Faridi, K., Joober, R. & Malla, A. Medication adherence mediates the impact of sustained cannabis use on symptom levels in first episode psychosis. Schizophrenia Research, 141:78-82, 2012.

Mueser, K., Gingerich, S. Treatment of co-occurring psychotic and substance use disorders. Social Work Public Health, 28(3-4):424-39. 2013, doi: 10.1080/19371918.2013.774676.

Sheidow, A., McCart, M., Zajac, K. & Davis, M. Prevalence and impact of substance use among emerging adults with serious mental health conditions. Psychiatric Rehabilitation Journal, 35(3):235-43, 2012.

Editorial consensus.

How might this affect my usual activities?

Continue antipsychotic medications

Harrow, M., Grossman, L., Jobe, T. & Herbener, E. Do patients with schizophrenia ever show periods of recovery? A 15 year multi-follow-up study. Schizophrenia Bulletin, 31(3):2145-2155, 2005.

Adjust antipsychotic medications

Uchida, H., Suzuki, T., Takeuchi, H., Arenovich, T. & Mamo, D. Low dose vs standard dose of antipsychotics for relapse prevention in schizophrenia: Meta-analysis. Schizophrenia Bulletin, 37(4):788-799, 2009.

Buchanan, R., Kreyenbuhl, J. et al The 2009 schizophrenia PORT psychopharmacological treatment recommendations and summary statements. Schizophrenia Bulletin, 36(1):71-93, 2010.

Wunderink, L., Nieboer, R, Wiersma, D., Sytema, S. & Nienhuis, F. Recovery in remitted first episode psychosis at 7 years of follow-up of an early dose reduction/discontinuation or maintenance treatment strategy. Journal of the American Medical Association - Psychiatry, 70(9):913-920, 2013.

Stop antipsychotic medications

Wunderink, L., Nieboer, R, Wiersma, D., Sytema, S. & Nienhuis, F. Recovery in remitted first episode psychosis at 7 years of follow-up of an early dose reduction/discontinuation or maintenance treatment strategy. Journal of the American Medical Association - Psychiatry, 70(9):913-920, 2013.

Viguera, A., Baldessarini, R., Hegarty, J., van Kammen, D. & Tohen, M. Clinical risk following abrupt and gradual withdrawal of maintenance neuroleptic treatment. Archives of General Psychiatry, 54(1):49-55, 1997.

Zipursky, R., Menezes, N. & Streiner, D. Risk of symptom recurrence with medication discontinuation in first-

episode psychosis: A systematic review. Schizophrenia Research,152:408-414, 2014.

Fraser, R., Berger, G., Killackey, E. & McGorry, P. Emerging psychosis in young people – part 3. Australian Family Physician, 35(5):329-332, 2006.

Saloman, C. & Hamilton, B. Antipsychotic discontinuation syndromes: A narrative review of the evidence and its integration into Australian mental health nursing textbooks. International Journal of Mental Health Nursing, 23:69-78, 2014.

Editorial consensus.

Editors: David Shern, Glyn Elwyn, Julie Kreyenbuhl, Robert Drake, Patricia Deegan, Lisa Dixon, Anthony Lehman, William Torrey, Manish Mishra, Yaara Zisman-Ilani
ISBN: 978-1-941487-17-4
License: CC BY-NC-ND 4.0 (International)
More information: http://optiongrid.org
